# Value of information analyses for advanced cell therapies: a systematic review

**DOI:** 10.1186/s13561-026-00728-w

**Published:** 2026-02-17

**Authors:** Alexander Heaps, Sean P. Gavan

**Affiliations:** 1 Manchester Centre for Health Economics, Division of Population Health, Health Services Research and Primary Care, School of Health Sciences, Faculty of Biology, Medicine and Health, The University of Manchester, 4.309 Jean McFarlane Building, Oxford Road, Manchester, M13 9PL UK; 2 Medical Student, Division of Medical Education, School of Medical Sciences, Faculty of Biology, Medicine and Health, The University of Manchester, Manchester, M13 9PL UK

**Keywords:** Advanced therapy medicinal product, ATMP, Cell therapy, Decision uncertainty, Expected value of perfect information, Systematic review, Value of information

## Abstract

**Background:**

Advanced cell therapies often face high parameter uncertainty at launch, prompting calls to collect further data about long-term effectiveness and safety. However, the value of collecting these data to support resource allocation decision-making is not known. Therefore, this study aimed to appraise all published value of information analyses for advanced cell therapies.

**Methods:**

A systematic review (PROSPERO: CRD42023446874) identified value of information analyses for advanced cell therapies between inception and 14 May 2025 (databases: Medline; Embase). Included studies reported the expected value of perfect information (EVPI), expected value of partial perfect information (EVPPI), expected value of sample information (EVSI) or expected net benefit of sampling (ENBS). Study design and value of information results were summarised in a narrative synthesis. Quality of reporting was assessed using the Consolidated Health Economic Evaluation Reporting Standards Value of Information (CHEERS-VOI) checklist.

**Results:**

Three published value of information analyses were identified: tisagenlecleucel for relapsed/refractory acute lymphoblastic leukemia; tisagenlecleucel for relapsed/refractory diffuse large B-cell lymphoma, and tumor infiltrating lymphocyte cell therapy for advanced melanoma. The beneficiary populations were 6, 36, and 400 individuals per year, respectively. All studies reported EVPI; two studies reported EVPPI. Estimated base case population EVPI was: €314,455, €0, and €2,250,000, respectively. Estimated EVPPI indicated that input parameters for survival extrapolations were the most valuable targets for further research specifically during scenario analyses that explored a lower cost of treatment acquisition.

**Conclusions:**

Value of information analyses will help decision-makers, analysts, and manufacturers understand whether long-term data collection is worthwhile to reduce decision uncertainty for advanced cell therapies at launch. Current estimates indicated that the value of further research is likely to be low. The value of collecting additional data will likely increase if future advanced cell therapies are priced such that their corresponding incremental cost-effectiveness ratio aligns with a relevant cost-effectiveness threshold and if they are indicated for larger beneficiary populations.

**Supplementary Information:**

The online version contains supplementary material available at 10.1186/s13561-026-00728-w.

## Introduction

High-cost advanced cell therapies are entering standard of care within healthcare systems internationally [1–5]. A key challenge facing decision-makers and health technology assessment agencies when appraising the value of advanced cell therapies is how best to handle the extent of uncertainty around input parameters for health outcomes [6–10]. To support resource allocation decision-making, further data collection is often suggested as one way to reduce this parameter uncertainty [11–13]. However, data collection is costly and the value of collecting these additional data either pre- or post-adoption for advanced cell therapies is not clear. A better understanding of whether the expected benefit achieved from further data collection outweighs its anticipated cost will help decision-makers navigate these calls to invest in collecting additional data on the short-term and long-term effectiveness of advanced cell therapies.

Advanced cell therapies are a type of advanced therapy medicinal product (ATMP) which typically aim to deliver restorative health gains after a single dose administration [14]. Examples of advanced cell therapies include chimeric antigen receptor-T (CAR-T) cell therapy [15], tumour infiltrating lymphocyte (TIL) cell therapy [16], and natural killer cell therapy [17]. Most examples available to date are autologous cell therapies which modify or enhance a patient’s own cells to produce the active treatment [18], with allogeneic cell therapies under active development [19]. Current licenced indications for advanced cell therapies are typically populations with severe disease that have not responded to earlier lines of treatment. However, as the capacity to deliver ATMPs begins to scale across healthcare systems, there is an expectation that the number of patient populations managed by advanced cell therapies will increase and the scope of conditions amenable to advanced cell therapies will begin to expand [20].

Establishing the cost-effectiveness of advanced cell therapies is often challenging because of the high upfront cost of treatment (in the region of $400,000 to $500,000 per administration [21]) and the high uncertainty around treatment effectiveness or long-term safety [22]. Parameter uncertainty in the magnitude and persistence of relative treatment effects over a full lifetime are often key drivers of cost-effectiveness. These parameters are also difficult to quantify because the clinical evidence available at launch is typically obtained from small early-phase single-armed trials [23–25]. Developments in flexible survival analysis methods, embedded within decision-analytic modelling, provide an appropriate means to handle these data challenges during health technology assessment for emerging treatments offering curative intent [26]. However, the magnitude of parameter uncertainty for new advanced cell therapies is unavoidable and is often observed in parallel with calls from different stakeholders to collect additional data.

Further data collection is valuable from a decision-maker’s perspective because it reduces decision uncertainty, expressed as the probability and consequence of recommending a strategy that is not cost-effective [27]. Collecting additional data can be implemented by delaying adoption decisions or by collecting longer-term follow-up data through an ‘only with research’ coverage agreement [28]. However, data collection incurs a cost and decision-makers must prioritise generating evidence for parameters that are expected to provide the most value [29]. Value of information analysis provides a suite of methods to help inform decisions to invest in further research [27, 30]. The expected value of perfect information (EVPI) provides a necessary condition for whether further data collection is valuable, the expected value of partial perfect information (EVPPI) helps to prioritise targets for further data collection, the expected value of sample information (EVSI) helps to guide future study design characteristics (for example, sample size or number of arms), and the expected net benefit of sampling (ENBS) compares the cost of future study designs against their expected benefit [30].

In the context of advanced cell therapies, where there is high uncertainty over longer-term health outcomes following treatment administration, value of information analyses can be highly beneficial to guide whether calls for further data collection are justified. Yet the extent to which value of information analyses for advanced cell therapies have been performed to date is not clear. A critical assessment of these value of information analyses will help to establish the conditions under which further data collection may be worthwhile for advanced cell therapies, and to help guide analysts and decision-makers when performing or interpreting similar studies in the future. Therefore, the aim of this study was to appraise all published value of information analyses for advanced cell therapies.

## Method

This study is a systematic review of all published value of information analyses for advanced cell therapies. The study is reported according to the Preferred Reporting Items for Systematic Reviews and Meta-analyses (PRISMA) 2020 statement [31]. The study was registered in advance via the International Prospective Register of Systematic Reviews (PROSPERO, identification number: CRD42023446874).

### Inclusion criteria

The review included any published full text value of information analyses for an advanced cell therapy indicated for any patient population in a peer-reviewed journal. Value of information analyses were defined as studies that reported either of the following results: EVPI, EVPPI, EVSI, or ENBS. For inclusion in this review, advanced cell therapies were required to have received regulatory approval or be in active development. The comparator strategy could be any relevant alternative management strategy. Studies that evaluated hypothetical treatments, tissue-engineered products, in-vivo gene therapies, were not written in English, or were reported as conference abstracts were excluded.

### Search strategy

Medline and Embase were searched electronically via the Ovid platform from inception until 14 May 2025. These two databases were sufficient because the majority of health economic evaluations can be found within their records [32]. The search strategy comprised free-text terms for value of information analyses and advanced cell therapies (reported in Supplementary Appendix 1). Terms for advanced cell therapies comprised generic terms for ATMPs and specific terms (branded and international non-proprietary) for treatments approved by the US Food and Drug Administration [33].

### Study identification

Two authors (AH, SPG) screened the abstracts against the inclusion criteria independently. Disagreements at this stage were resolved by progressing the study to full text review. The extent of agreement was determined by Cohen’s kappa [34]. Candidate studies were read in full by two authors (AH, SPG) independently after abstract screening to decide whether they should be included. Disagreements at this stage were resolved during one face-to-face meeting between the authors. The reference lists of included studies were screened to identify additional studies that met the inclusion criteria. No automation tools were used for study identification.

### Data extraction and analysis

The following data were extracted manually from the included studies by one author (AH): intervention strategy, comparator strategy, target population, country of the analysis, type of decision-analytic model used (if relevant), type of value of information analysis performed, size of the beneficiary population for population-level value of information analyses, reported value of information results (EVPI, EVPPI, EVSI, ENBS), and value of the cost-effectiveness threshold used to calculate net monetary benefits. Extracted data were verified by a second author (SPG).

The extracted data were summarised in a table. Descriptive statistics summarised the characteristics of the included studies (target population; decision-making jurisdiction; proportion reporting either EVPI, EVPPI, EVSI, or ENBS). Each included study was then analysed individually as part of a narrative synthesis. The narrative synthesis evaluated each study according to the methods used, source of input parameter data, value of information results, and key parameters for further research if reported. Subgroup analyses to explore heterogeneity between the included studies was not performed.

### Quality assessment

The quality of the included studies was assessed by the value of information-specific update to the Consolidated Health Economic Evaluation Reporting Standards 2022 (CHEERS-VOI) statement [35]. The included studies were assessed by one author (AH) against the twenty-eight criteria for reporting methods and results (reported in full, partially, or not at all). A second author (SPG) verified these quality assessment decisions.

## Results

**Fig. 1:**
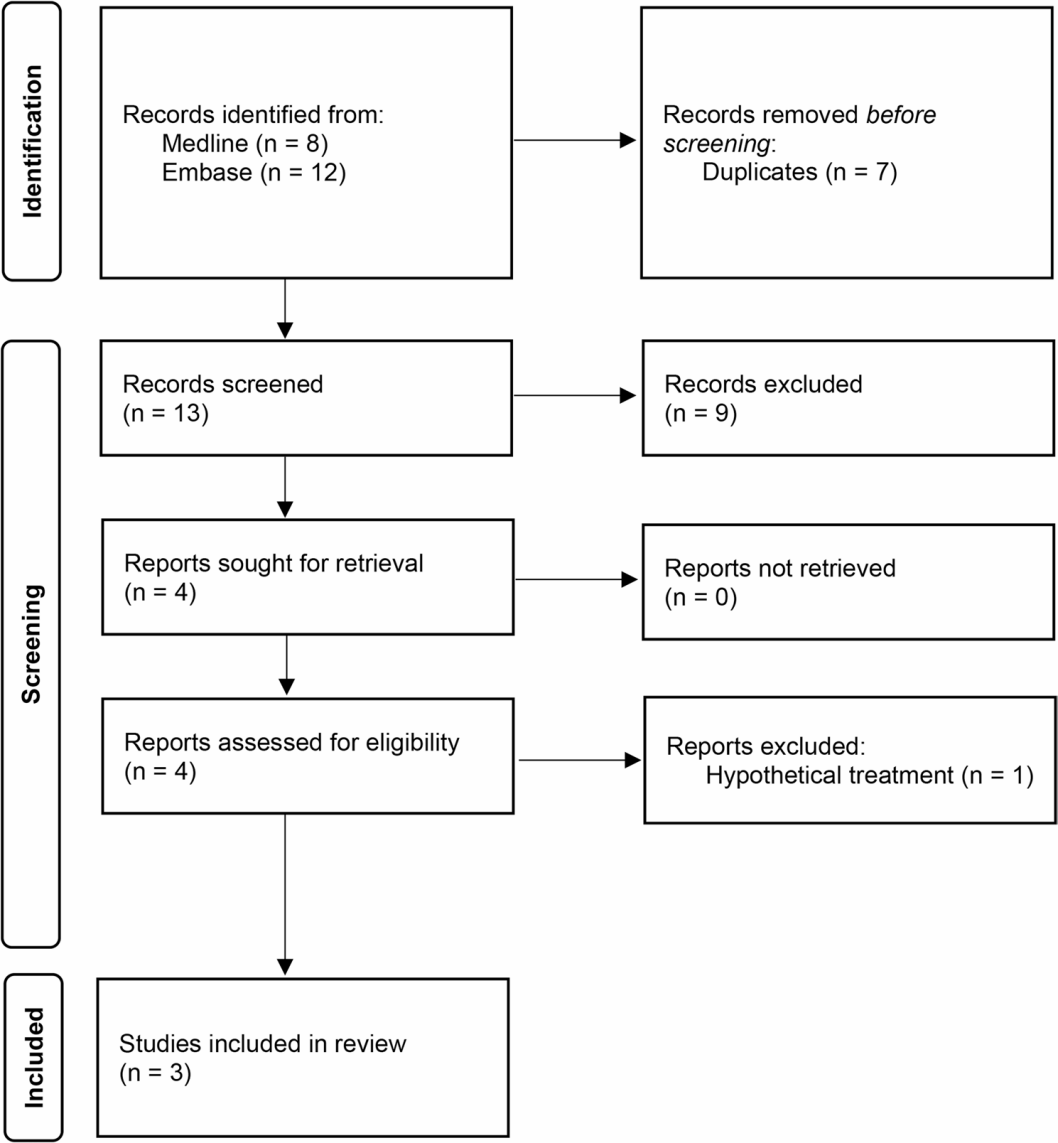
PRISMA flow diagram of included studies. Abbreviations: PRISMA: Preferred Reporting Items for Systematic reviews and Meta-Analyses [31]

Figure 1 reports the PRISMA flow diagram to illustrate how studies were identified for inclusion in this review [31]. The database search identified thirteen titles and abstracts to screen. The two reviewers were in perfect agreement with regards to screening decisions (Cohen’s Kappa: 1). The final sample comprised three published value of information analyses for advanced cell therapies [36–38]. These studies are summarised in Table 1.

**Table 1:** Summary of included studies

Author (Year)	Country	Population	Intervention	Comparator	VOI Method	Beneficiary Population	Population VOI Result
Carey et al. (2022) [36]	Ireland	Relapsed/refractory paediatric acute lymphoblastic leukaemia	Tisagenlecleucel (Kymriah^®^)	Blinatumomab (Blincyto^®^)	EVPI; EVPPI	6 patients per year	**Base case** EVPI: €314,455 **Acquisition Cost Sensitivity Analysis** EVPI: €1,149,810 EVPPI: €371,813 (survival analysis)
Carey et al. (2023) [37]	Ireland	Relapsed/refractory diffuse large B cell lymphoma	Tisagenlecleucel (Kymriah^®^)	Salvage chemotherapy	EVPI; EVPPI	36 patients per year	**Base case** EVPI: €0 **Acquisition Cost Sensitivity Analysis** EVPI: €3,989,438 EVPPI: €1,128,053 (survival analysis)
Retèl et al. (2018) [38]	The Netherlands	Advanced melanoma	TIL cell therapy	Ipilimumab	EVPI	400 patients per year	**Base Case** EVPI: €2,250,000

*Abbreviations*: *EVPI* expected value of perfect information, *EVPPI* expected value of partial perfect information, *TIL* tumor infiltrating lymphocyte, *VOI* value of information

### Descriptive analysis

All studies assessed advanced cell therapies for oncology indications (relapsed or refractory paediatric and young adult acute lymphoblastic leukaemia [36]; relapsed or refractory diffuse large B cell lymphoma [37]; and advanced melanoma [38]). The majority of studies (*n* = 2) were reported for the Irish healthcare system [36, 37]; one study was reported for the healthcare system in The Netherlands [38]. Two studies reported a value of information analysis for the CAR-T cell therapy tisagenlecleucel [36, 37]. One study reported their results for a TIL cell therapy in development [38]. All studies reported the population EVPI [36–38]. Two studies reported the population EVPPI [36, 37]. No study reported either the EVSI or ENBS.

### Value of information analyses

In 2022, Carey and colleagues [36] reported a cost-effectiveness and value of information analysis of tisagenlecleucel versus blinatumomab for relapsed/refractory paediatric acute lymphoblastic leukaemia from the perspective of the Healthcare Service Executive in Ireland. A 3-state partitioned survival analysis estimated cost-effectiveness over a lifetime time horizon. Effectiveness data for tisagenlecleucel were obtained from pooling two single-arm phase II trial data [39, 40]. Effectiveness data for blinatumomab were obtained from a single-arm phase I/II trial [41]. The acquisition cost of tisagenlecleucel was €301,762. At a cost-effectiveness threshold of €45,000 per quality-adjusted life year (QALY) gained, the probability that tisagenlecleucel was cost-effective at its list price was 16%. The population who would benefit from further research comprised 6 patients per year. The 10-year population EVPI was estimated as €314,455 in the base case analysis. In a sensitivity analysis, the list price of tisagenlecleucel was reduced so that the estimated incremental cost-effectiveness ratio (ICER) was equal to the cost-effectiveness threshold (€45,000 per QALY gained). At this reduced list price, the ten-year population EVPI was €1,149,810. In addition, an EVPPI analysis at this reduced list price found that uncertainty associated with the survival analysis input parameters was the most valuable target for further research (ten-year population EVPPI: €371,813).

In 2023, Carey and colleagues [37] reported a cost-effectiveness and value of information analysis of tisagenlecleucel versus salvage chemotherapy for relapsed/refractory diffuse large B cell lymphoma from the perspective of the Healthcare Service Executive in Ireland. A 3-state partitioned survival analysis estimated cost-effectiveness over a lifetime time horizon. Effectiveness data for tisagenlecleucel were obtained from a phase II single-armed trial of 115 patients [42]. Effectiveness data for salvage chemotherapy were obtained from pooling observational data on different chemotherapy regimens [43]. The acquisition cost of tisagenlecleucel was €301,762. At a cost-effectiveness threshold of €45,000 per QALY gained, the probability that tisagenlecleucel was cost-effective at its list price was 0%. The population who would benefit from further research comprised 36 patients per year. The 10-year population EVPI was estimated as €0 in the base case analysis. In a sensitivity analysis, the list price of tisagenlecleucel was reduced so that the estimated ICER was equal to the cost-effectiveness threshold (€45,000 per QALY gained). At this reduced list price, the ten-year population EVPI was €3,989,438. In addition, an EVPPI analysis at this reduced list price found that uncertainty associated with the survival analysis input parameters was the most valuable target for further research (ten-year population EVPPI: €1,128,053).

In 2018, Retѐl and colleagues [38] reported a cost-effectiveness and value of information analysis of TIL cell therapy versus ipilimumab for advanced melanoma from the perspective of the healthcare system in The Netherlands. A 3-state Markov model estimated cost-effectiveness over a ten-year time horizon. Effectiveness data for the TIL cell therapy were obtained from two single-arm phase II trials [44, 45]. Effectiveness data for ipilimumab were obtained from a phase III randomised controlled trial [46]. The acquisition cost of TIL cell therapy was reported as €62,000. At a cost-effectiveness threshold of €80,000 per QALY gained, the probability that the TIL cell therapy was cost-effective at its list price was 91%. The population who would benefit from further research comprised 400 patients per year. The population EVPI was approximately €2,250,000, however the time horizon over which further research was assumed to be valuable was not reported.

### Quality assessment

Table 2 reports the assessment of study quality against the criteria for the methods and results sections in the CHEERS-VOI statement [35]. On balance, the included studies are all broadly of good quality in relation to the frequency of reported items. The requirements for describing health economic analysis plans, distributional effects, and engagement with people affected by the study are likely underreported across the sample because these items were recent additions to the reporting criteria [47]. Of the two studies that estimated EVPPI [36, 37], the method to estimate these outcomes (gaussian process regression [48]) was reported clearly. Reporting about the potential biases in the evidence base (specifically the use of naïve indirect comparisons from single-armed studies) was variable across the sample, but handled effectively by each study with sensitivity analyses.

**Table 2:** Quality assessment of reported methods and results

CHEERS-VOI Reporting Criteria	Carey et al. (2022) [36]	Carey et al. (2023) [37]	Retèl et al. (2018) [38]
Methods			
Health Economic Analysis Plan	✖	✖	✖
Study Population	✔	✔	✔
Setting & Location	✔	✔	✔
Comparators	✔	✔	✔
Perspective	✔	✔	✔
Time Horizon	✔	✔	✔
Discount Rate	✔	✔	✔
Selection of Outcomes	✔	✔	✔
Measurement of Outcomes	✔	✔	✔
Valuation of Outcomes	✔	✔	✔
Measurement & Valuation of Resources & Costs	✔	✔	✔
Currency, Price Date, & Conversion	✔	✔	P
Rationale & Description of the Model	✔	✔	✔
VOI Estimation Methods	✔	✔	✔
Analytics & Assumptions	✔	✔	✔
Evidence Base	P	P	✔
Characterizing Heterogeneity	NA	NA	NA
Characterizing Distributional Effects	✖	✖	✖
Characterizing Uncertainty	✔	✔	✔
Parameters of Interest in VOI Analysis	✔	✔	✔
Study Design(s) Proposed in VOI Analysis	NA	NA	NA
Data Generation for EVSI	NA	NA	NA
Cost of Research Studies for ENBS	NA	NA	NA
Approach to Engagement with People Affected by the Study	✖	✖	✖
Results			
Study Parameters	✔	✔	✔
Summary of Main Results	✔	✔	✔
Effect of Uncertainty	✔	✔	✔
Effect of Engagement with People Affected by the Study	✖	✖	✖

Reporting criteria items from the CHEERS-VOI statement [35]

Notation: ✔: reported fully; P: reported partially; ✖: not reported; NA: not applicable

## Discussion

Decision-makers face regular appeals to collect further evidence on the effectiveness of advanced cell therapies. This study identified three published value of information analyses designed to quantify whether further data collection for advanced cell therapies is worthwhile. All studies reported the population EVPI, two studies reported the population EVPPI, and no study reported either the EVSI or ENBS. The reported estimates from these studies indicated that the expected benefit from further data collection was relatively low because the estimated population EVPI across all studies did not exceed around €2 million. A greater emphasis on quantifying the expected value of further data collection in the future will help decision-makers and analysts appraise if, and how, such data collection strategies should be undertaken for emerging advanced cell therapies within their indicated target populations and healthcare jurisdiction.

Understanding the size of the population who could benefit from further research is vital to quantify the value of further data collection [30]. The size of this beneficiary population comprises incident and prevalent patients alongside the duration over which further research will be valuable [29]. At present, the number of incident patients who will benefit from new advanced cell therapies is low because these treatments are typically reserved for later stages in pathways of care (such as populations who are refractory to earlier lines of treatment). From a policymaking perspective, lower estimates of incidence limit the expected population who can benefit from further research, all else being equal. The duration over which further research is valuable for advanced cell therapies is also uncertain. Whilst studies often use arbitrary time horizons for population value of information estimates (such as 10-years), analysts should consider providing justification for their chosen time horizon to avoid overestimating the size of future beneficiary populations in the context of the dynamic market for advanced therapy medicinal products.

The pricing policy for advanced cell therapies is also pivotal to determining the value of further research. It is common for cost-effectiveness analyses of advanced cell therapies to estimate ICERs in their base case that greatly exceed reported thresholds for cost-effectiveness [49]. These scenarios arise because the expected gain in quality-adjusted life years is far lower than the health opportunity cost of the advanced cell therapy. In such cases, if all parameter uncertainty was resolved, the impact on decision-making will likely be negligible (i.e. the treatment will never be cost-effective, even under conditions of perfect information, due to the high opportunity cost of treatment). As pricing policies bring estimated ICERs closer to relevant thresholds for cost-effectiveness, decision uncertainty will increase and, in turn, the expected benefit from further research will also increase [20]. As a practical step, analysts can explore this phenomenon in a scenario analysis by performing value of information analyses after reducing the acquisition cost of the advanced cell therapy until the estimated ICER meets the relevant cost-effectiveness threshold [36, 37]. An alternative approach is to explore how different payment models for advanced cell therapies affect the value of further research. For example, rather than a single upfront payment for treatment, payers are exploring the use of repeated payments conditional on patients achieving pre-specified outcomes to smooth the time profile of expenditures on ATMPs, and short-term outcome-based payments [20, 50, 51]. These alternative payment models will impact the value of further research by affecting the magnitude and/or uncertainty in the expected health opportunity cost.

Structural uncertainty is often considerable when designing model-based cost-effectiveness analyses of advanced cell therapies. In particular, analysts must decide on the most appropriate technique to extrapolate survival outcomes over a lifetime when faced with complex hazard functions driven by the hypothesised curative nature of treatment [52]. Flexible methods for survival analysis are now common to extrapolate health outcomes in cost-effectiveness analyses of advanced cell therapies (such as flexible spline models, mixture-cure models, and piecewise survival analysis) [26]. The plausibility of these extrapolations can be challenging to establish for first-in-class advanced cell therapies where longer-term follow-up data are unlikely to exist for the target patient population. Recent developments in expert elicitation methods for temporal changes in treatment effects may be one approach to improve prior estimates of health outcomes in the absence of long-term data [53]. Alternatively, if external data sources describing longer-term outcomes from treatment with an advanced cell therapy are available, then techniques to leverage these external data to support survival analysis extrapolations can be used [54]. Analysts could also consider using matching-adjusted indirect comparisons where plausible to improve estimates of comparative effectiveness for advanced cell therapies relative to using naïve indirect comparisons. Understanding how alternative methods to estimate long-term effectiveness affect value of information results would be a worthwhile activity for analysists evaluating future examples of advanced cell therapies.

Analysts who wish to use value of information methods to help decision-makers with research recommendations for advanced cell therapies can build on the studies identified by this review. Population EVPPI is crucial to identify the most valuable parameters for further data collection. Advanced cell therapies are often adopted with a managed entry agreement to facilitate prospective data collection [55]. Therefore, EVPPI can inform the design of managed entry agreements to ensure that data are collected which will help support decision-making [56]. No study reported the EVSI or ENBS which assess the benefit and value of alternative study designs, respectively [30]. EVSI and ENBS for advanced cell therapies would help to demonstrate the value of specific research designs to external research funding bodies (for example, longer-term health utility measurement or a cohort study for adverse event surveillance). To support these analyses in the future, a greater emphasis on the resources and costs required to deliver research studies using advanced cell therapies would be valuable given the known challenges to study design and delivery constraints with ATMPs [57, 58].

One limitation of this review was that the search strategy only identified value of information analyses by whether key terms were stated in the title or abstract. This approach may have omitted studies if value of information analyses were not reported in these two sections. However, this technique has been used effectively by previous reviews to detect value of information analyses across a range of health conditions [59, 60]. The decision to include English language studies only may have resulted in some relevant non-English studies being excluded. A second limitation was that hypothetical advanced cell therapies were not part of the review inclusion criteria. Whilst evaluations of hypothetical interventions can offer insights into early product development decisions [61, 62], this review focussed on addressing the downstream challenges faced by decision-makers who are tasked with producing recommendations about actual health technologies in routine care based on current levels of evidence. A third limitation was that the CHEERS-VOI statement [35], used to assess the quality of reporting, was published after the included studies were published. This may explain why some criteria were reported incompletely.

Future research should aim to undertake value of information analyses alongside cost-effectiveness analyses for advanced cell therapies if concerns about parameter uncertainty are relevant to decision-makers. Guidance for performing value of information analyses is emerging and reporting standards are now available [35, 63–65]. Future research could also investigate how parameter uncertainty from alternative techniques to estimate survival outcomes for curative therapies (for example, piecewise survival analysis, spline models, mixture-cure models) affects the value of further research in addition to expected cost-effectiveness. Finally, future research could investigate practical strategies to support further data collection for advanced cell therapies, and ATMPs more generally, by interviewing key stakeholders within the healthcare system (including health technology assessment decision-makers, payers, providers, manufacturers, and patient organisations).

## Conclusion

High-cost advanced cell therapies typically have extensive parameter uncertainty at launch prompting health technology assessment agencies, decision-makers, and care providers to consider whether further data collection is worthwhile. Collecting additional data to reduce parameter uncertainty may be valuable in this context, but few value of information analyses for advanced cell therapies have been performed to date. The existing evidence base indicates that the value of further data collection is relatively low for current examples of advanced cell therapies, driven by high treatment acquisition costs and low incidence rates for beneficiary patient populations. A systematic approach to evaluating the costs and benefits of data collection strategies in the future will ultimately help to guide patient access to forthcoming advanced cell therapies in the face of considerable decision uncertainty.

## Supplementary Information

Supplementary Material 1.

## Data Availability

All data generated or analysed during this study are included in this published article.

## Abbreviations

ATMP: Advanced therapy medicinal product
CAR-T: Chimeric antigen receptor-T
CHEERS-VOI: Consolidated Health Economic Evaluation Reporting Standards Value of Information
ENBS: Expected net benefit of sampling
EVPI: Expected value of perfect information
EVPPI: Expected value of partial perfect information
EVSI: Expected value of sample information
ICER: Incremental cost-effectiveness ratio
PRISMA: Preferred Reporting Items for Systematic Reviews and Meta-analyses
PROSPERO: International Prospective Register of Systematic Reviews
QALY: Quality-adjusted life year
TIL: Tumour infiltrating lymphocyte
