# Supplementary material for: Value of information analyses for advanced cell therapies: a systematic review
Source: Health Econ Rev. 2026 Feb 17;16:36. doi: 10.1186/s13561-026-00728-w (PMC13014996; doi:10.1186/s13561-026-00728-w)
Supplement: Supplementary file 1 — Supplementary Material 1. [file 13561_2026_728_MOESM1_ESM.pdf]

Value of Information Analyses for Advanced Cell Therapies: A Systematic Review

Supplementary Appendix

Medline and Embase Electronic Search Strategy

1 Advanced Therapy Medicinal Product*.mp
2 ATMP*.mp
3 Cell Therap*.mp
4 Gene Therap*.mp
5 CAR-T.mp
6 Chimeric Antigen Receptor*.mp
7 Adoptive Cell Therap*.mp
8 Tumour Infiltrating Lymphocyte*.mp
9 Tumor Infiltrating Lymphocyte*.mp
10 TIL.mp
11 or/1-10
12 Yescarta.mp
13 Laviv.mp
14 Zynteglo.mp
15 Tecartus.mp
16 Carvykti.mp
17 Skysona.mp
18 Abecma.mp
19 Breyanzi.mp
20 Omisirge.mp
21 Provenge.mp
22 Kymriah.mp
23 Amtagvi.mp
24 Aucatzyl.mp
25 Casgevy.mp
26 Encelto.mp
27 Lantidra.mp
28 Lenmeldy.mp
29 Libmeldy.mp
30 Lyfgenia.mp
31 Ryoncil.mp
32 Tecelra.mp
33 axicabtagene ciloleucel.mp
34 azficel-t.mp
35 betibeglogene autotemcel.mp
36 brexucabtagene autoleucel.mp
37 ciltacabtagene autoleucel.mp
38 elivaldogene autotemcel.mp
39 idecabtagene vicleucel.mp
40 lisocabtagene maraleucel.mp
41 omidubicel-onlv.mp
42 sipuleucel-t.mp
43 tisagenlecleucel.mp

44 Lifileucel.mp
45 obecabtagene autoleucel.mp
46 exagamglogene autotemcel.mp
47 revakinagene taroretcel*.mp
48 donislecel.mp
49 atidarsagene autotemcel.mp
50 lovotibeglogene autotemcel.mp
51 remestemcel*.mp
52 afamitresgene autoleucel.mp
53 or/12-52
54 Value of Information.mp
55 Expected Value of Perfect Information.mp
56 Expected Value of Partial Perfect Information.mp
57 Expected Value of Samp* Information.mp
58 EVPI.mp
59 EVPPI.mp
60 EVSI.mp
61 or/54-60
62 11 or 53
63 61 and 62
64 remove duplicates from 63
